# Cholera Infantum

**Published:** 1900-07

**Authors:** A. E. Chatfield

**Affiliations:** Cleveland, O.


### CHOLERA INFANTUM.

BY A. E. CHATFIELD, M.D.,

CLEVELAND, O.

No subject is more important, especially at this period of the year, than that of infantile intestinal disorders, usually caused by the intense heat of summer, dentition, improper diet, and unsanitary surroundings. As physicians we are called upon to combat that disease so dreaded by mothers—cholera infantum, it generally occurring in the poorer classes where, alas! so often such a thing as asepsis is unknown.

I wish to report to you some cases that I have been treating in which I have used with great success glyco-thymoline (Kress).

Aggie McK., aged 14 months, cutting four teeth, taken suddenly sick in the night with colicky pains, vomiting and purging, pulse 140, temperature 104 degrees; the stools in a few hours becoming copious, musty odor, greenish in character. The treatment at that time was mustard paste to abdomen and mild purgative. The next day I prescribed standard drugs, but the bowel trouble did not abate; she kept on the same manner, growing weaker and gradually emaciated. On the fourth day commenced glyco-thymoline (Kress) with equal parts liq. bismuth, teaspoonful every two hours. It acted like a charm. After two doses could notice a change for the better, and in three days the child was convalescent.

Walter S., four months old; delicate, nervous, irritable from birth; had been suffering with cholera infantum for over a week when I was called to see him; he had wasted to a mere nothing—eyes sunken, semi-comatose, skin clammy, bowels moving every few minutes; ordered an enema of warm water with one ounce of glyco-thymoline (Kress) to pint. Administered internally

| | |
|---|---|
| Bismuth subcarbonas | dr. i. |
| Spts. myristicæ | min. xx. |
| Spts. vini gallici | dr. iii. |
| Glyco-thymoline (Kress) | oz. ½. |
| Mistura creta, q. s. a. d | oz. iii. |

M. Sig.: Teaspoonful every three hours.

The next morning when I saw him there was a slight change for the better and the bowels were not so active. Same treatment was kept up, there was a gradual recovery in three or four days, the stools were normal, and other symptoms had disappeared. I sent him to the fresh-air camp, giving the mother a bottle of glyco-thymoline (Kress), with directions to use one-half teaspoonful, diluted, three times a day. The child is picking up nicely.

John T., two-months-old baby. Typical case of cholera infantum, had small hopes of saving the little one. Put him on equal parts of liq. bismuth and glyco-thymoline (Kress), one-half teaspoonful doses every three hours. It controlled the vomiting and regulated the bowels, and the child made a nice recovery.

I have also used glyco-thymoline (Kress) in syphilitic sore mouth, ulcerative stomatitis and hemorrhoids, and find it a splendid palliative. The results obtained were entirely satisfactory, both to myself and patients.

I shall continue to use it in my practice.

*August 7th, 1899.*

---

### PINEAPPLE FOR CROUP.

Don't forget to have a jar of strong pineapple syrup in a glass can on one of the top shelves, so that if there is a sign of croup pineapple can be instantly given. Pineapple juice is excellent for croup and diphtheria if taken in season. Don't wait until the disease comes, and then long for pineapple juice, but can them when you can get the pineapples, and have it all ready for an emergency. A few teaspoonfuls of the juice at a time is the dose; and then wait, and in an hour or two give some more.—*Exchange.*

